# Supplementary material for: A repurposed AMP binding domain reveals mitochondrial protein AMPylation as a regulator of cellular metabolism
Source: Nat Commun. 2025 Aug 23;16:7863. doi: 10.1038/s41467-025-63014-z (PMC12375108; doi:10.1038/s41467-025-63014-z)
Supplement: Supplementary file 1 — Supplementary Information [file 41467_2025_63014_MOESM1_ESM.pdf]

A repurposed AMP binding domain reveals mitochondrial protein AMPylation as a regulator of cellular metabolism

Abner Gonzalez[1], Alex Pon[1], Kelly Servage[2,3], Krzysztof Pawłowski[2], Yan Han[4], and Anju Sreelatha [1,5] *

Supplementary Figures S1-S18

Supplementary Tables 1-3

Figure S1

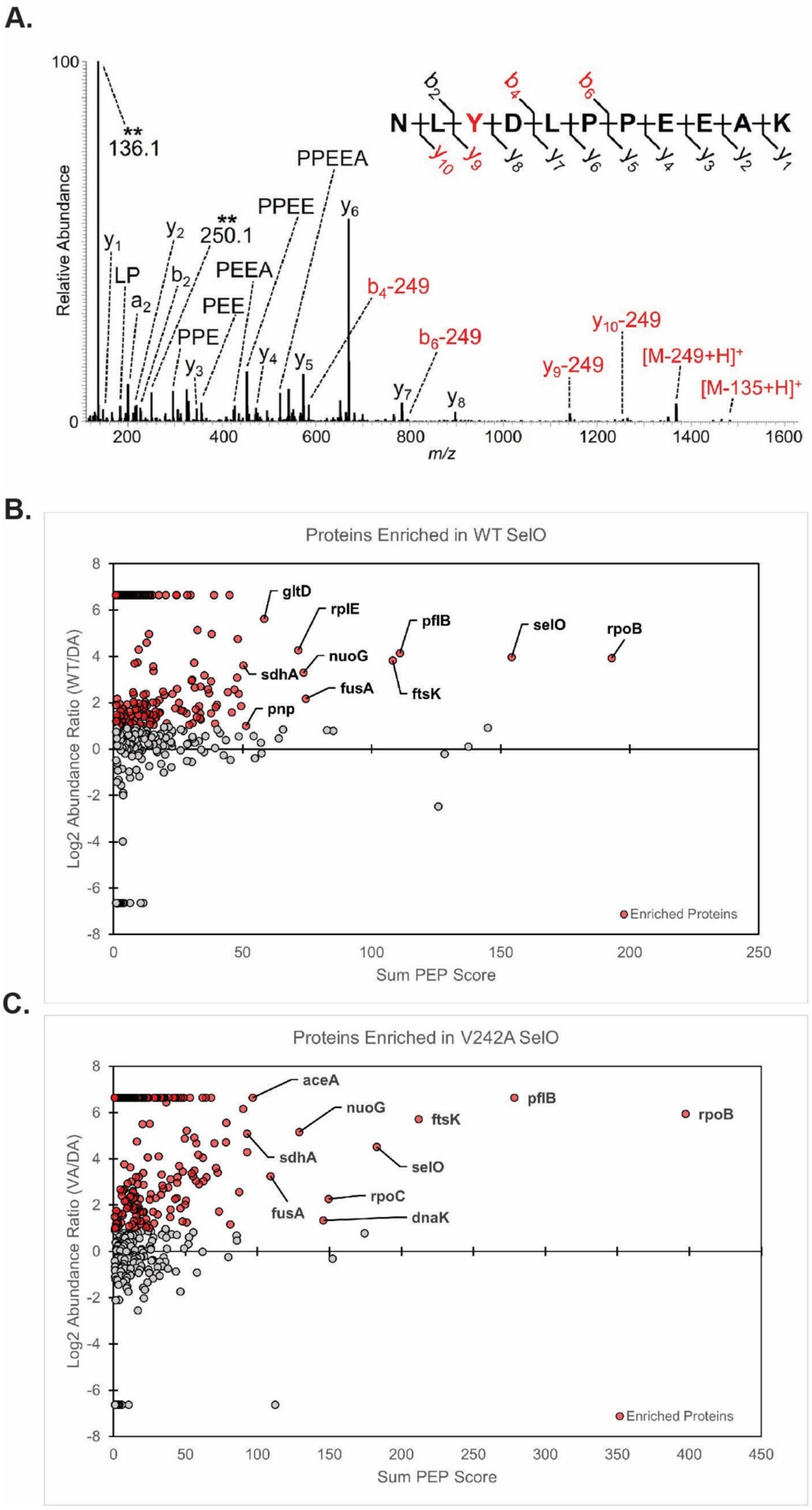

A.
100
0
Relative Abundance
m/z
200
400
600
800
1000
1200
1400
1600
** 136.1
** 250.1
N L Y D L P P E E A K
b_2 b_4 b_6
y_{10} y_9 y_8 y_7 y_6 y_5 y_4 y_3 y_2 y_1
PPEEA
PPEE
PEEA
PEE
PPE
LP
y_1
y_2
b_2
a_2
y_3
y_4
y_5
y_6
y_7
y_8
b_4-249
b_6-249
y_9-249
y_{10}-249
$[M-249+H]^+$
$[M-135+H]^+$
B.
Proteins Enriched in WT SelO
Log2 Abundance Ratio (WT/DA)
Sum PEP Score
gltD
rplE
pflB
selO
rpoB
nuoG
sdhA
fusA
ftsK
pnp
Enriched Proteins
C.
Proteins Enriched in V242A SelO
Log2 Abundance Ratio (VA/DA)
Sum PEP Score
aceA
nuoG
pflB
rpoB
ftsK
sdhA
selO
rpoC
fusA
dnaK
Enriched Proteins

Supplementary figure 1. GlnA is AMPylated on Tyr-398.

(A) MS/MS spectrum of AMPylated tryptic peptide ion NLYDLPPEEAK from GlnA protein. The precursor $[M+2H]^{2+}$ ion (*m/z* 809.35) was subjected to HCD fragmentation to generate the spectrum shown and the AMPylation site was localized to the tyrosine residue highlighted in red. Fragment ions containing the AMPylated residue (red) show characteristic mass shifts corresponding to partial loss of the AMP group (-249 and -135 Da). Unique ions corresponding to neutral loss of the AMP group are also present at 136.1 and 250.1 Da (labeled with **).

(B-C) Smear plots of the full set of proteins identified by label-free quantitative MS/MS analysis. Red dots denote enriched proteins in WT SelO (B) or V242A SelO (C) over DA SelO. Sum PEP Score represents protein ion scores. The top 10 most abundant enriched proteins from each dataset are labeled.

Figure S2

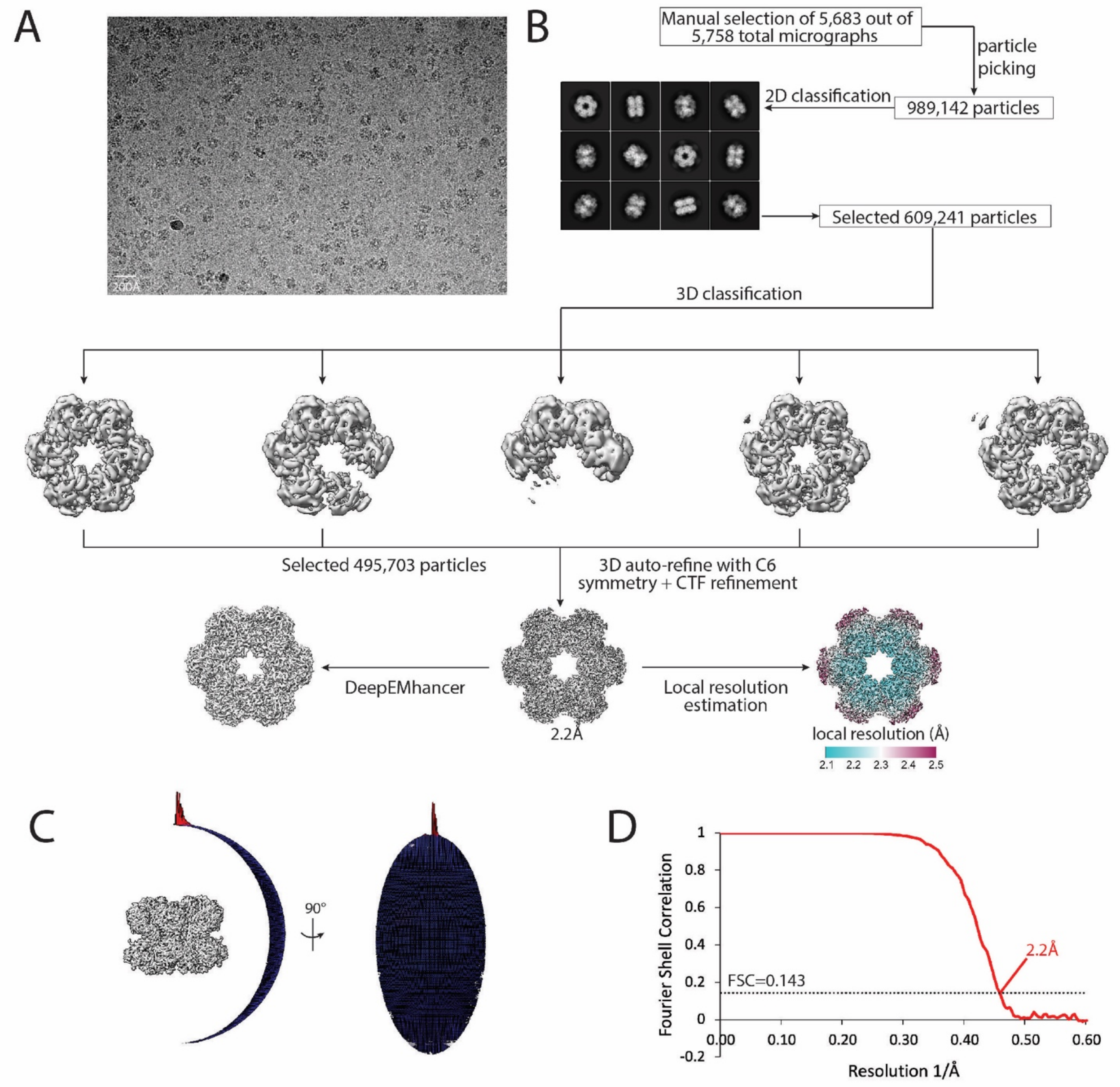

Supplementary figure 2. Cryo-EM data processing for GlnA.

(A) Representative cryo-EM raw micrograph.

(B) Schematic showing cryo-EM data processing steps for obtaining the 3D reconstruction of the GlnA dodecamer complex. Representative 2D class averages and the local resolution map are also shown.

(C) Euler angle distribution plot for the GlnA dodecamer complex.

(D) Fourier Shell Correlation (FSC) plot for the GlnA dodecamer complex.

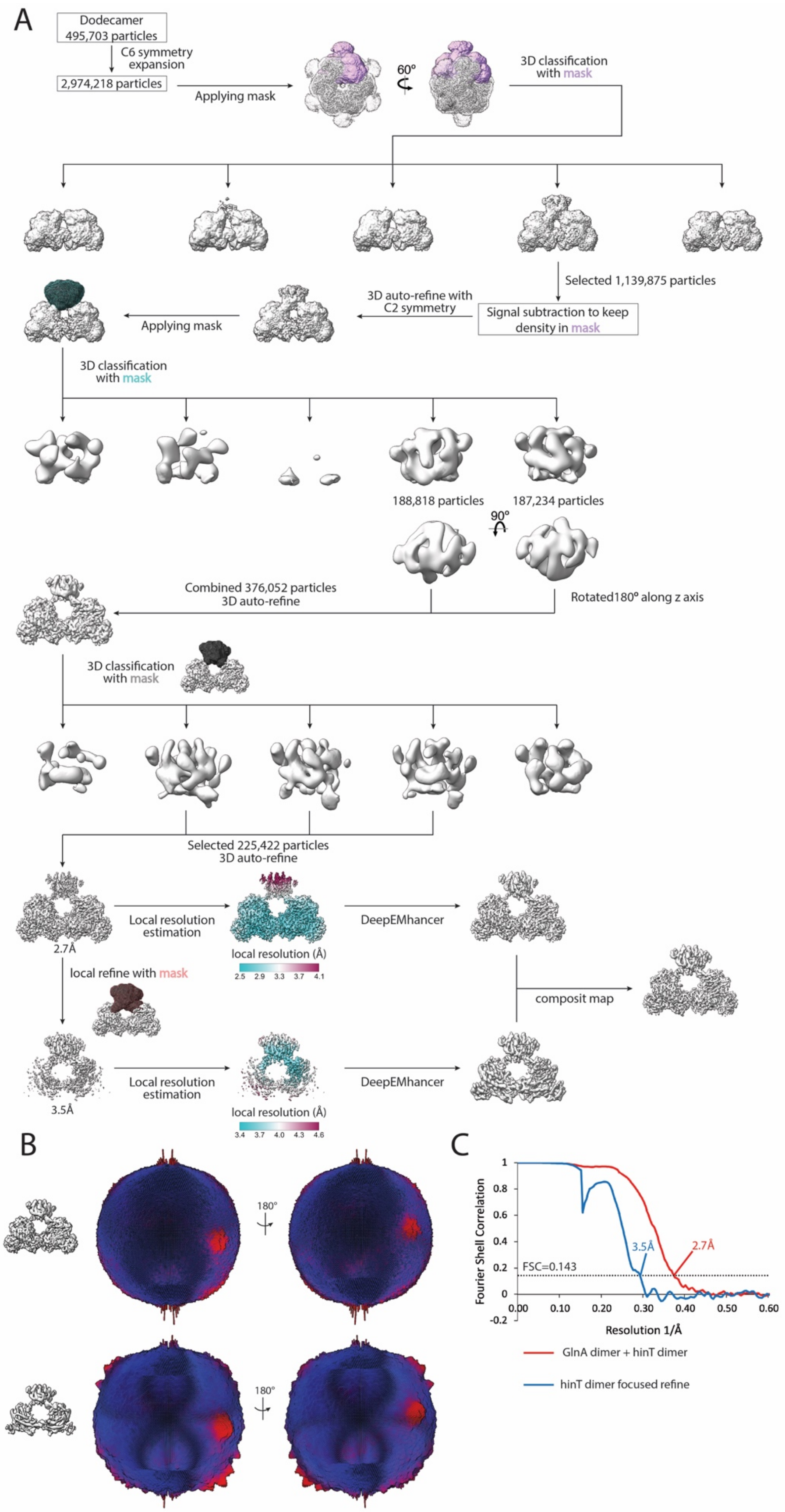

A
Dodecamer
495,703 particles
C6 symmetry expansion
2,974,218 particles
Applying mask
60°
3D classification with mask
Selected 1,139,875 particles
Signal subtraction to keep density in mask
3D auto-refine with C2 symmetry
Applying mask
3D classification with mask
188,818 particles
187,234 particles
90°
Rotated180° along z axis
Combined 376,052 particles 3D auto-refine
3D classification with mask
Selected 225,422 particles 3D auto-refine
2.7Å
Local resolution estimation
local resolution (Å)
2.5 2.9 3.3 3.7 4.1
DeepEMhancer
local refine with mask
composit map
3.5Å
Local resolution estimation
local resolution (Å)
3.4 3.7 4.0 4.3 4.6
DeepEMhancer
B
180°
180°
C
Fourier Shell Correlation
Resolution 1/Å
FSC=0.143
3.5Å
2.7Å
1 0.8 0.6 0.4 0.2 0 -0.2
0.00 0.10 0.20 0.30 0.40 0.50 0.60
GlnA dimer + hinT dimer
hinT dimer focused refine

Supplementary figure 3. Cryo-EM data processing for GlnA bound with hinT.

- **(A)** Schematic showing cryo-EM data processing steps for obtaining the 3D reconstruction of the GlnA dimer with hinT H101N.
- **(B)** Euler angle distribution plot for the GlnA dimer with hinT H101N (upper) and the locally refined hinT H101N (lower).
- **(C)** Fourier Shell Correlation (FSC) plot for the GlnA dimer with hinT H101N (red) and the locally refined hinT H101N (blue).

Figure S4

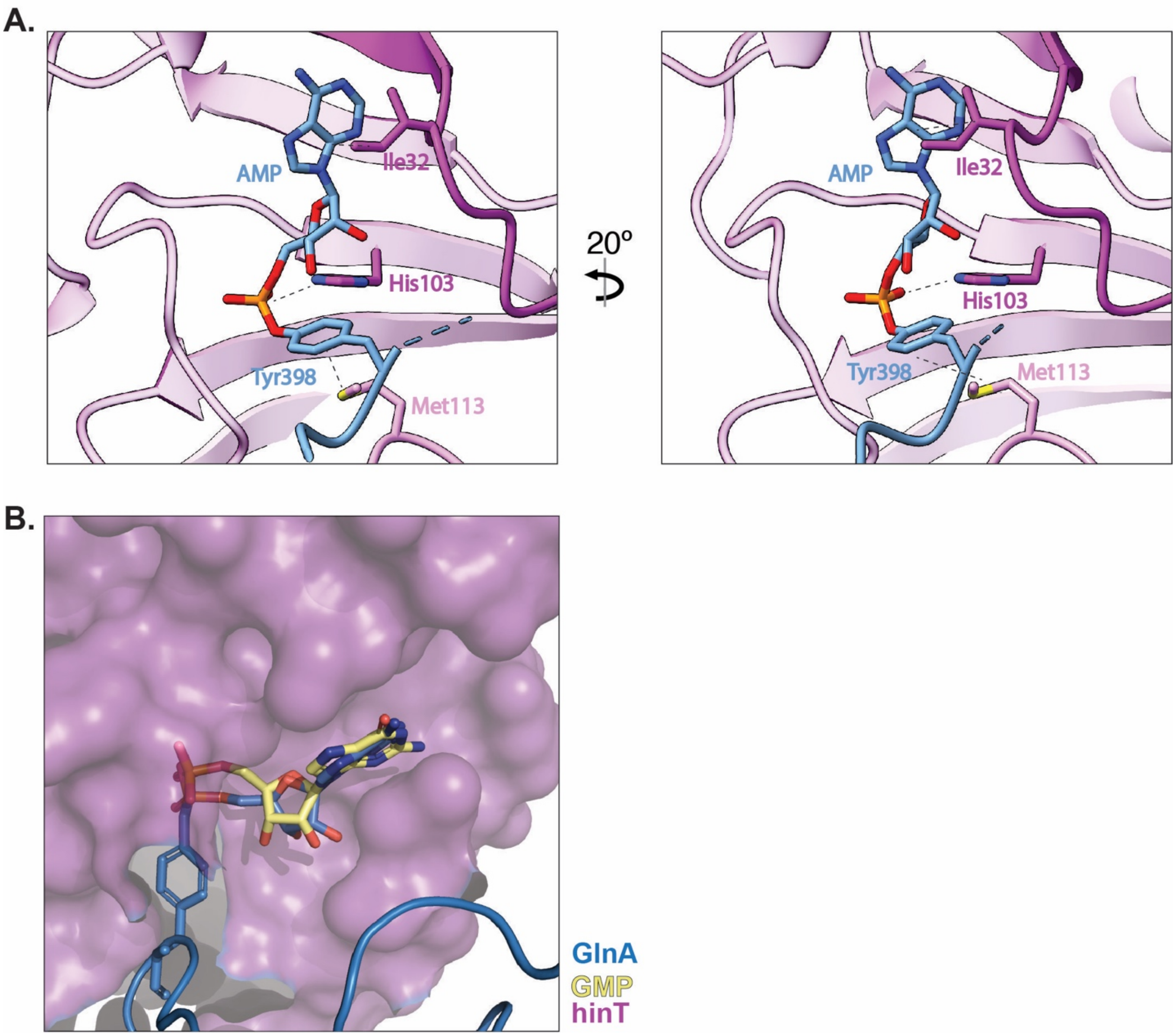

Supplementary figure 4. Amino acid residues in the hinT active site facilitate binding of nucleotide and AMPylated protein. Related to Figure 2.

(A) Enlarged image of the nucleotide binding pocket of hinT H101N highlighting the hinT amino acid interactions with AMPylated Tyr398 of GlnA.

(B) Stick representation of superimposed GMP from hinT H101A (PDB: 3N1T) with GlnA Y398-AMP in the binding pocket of hinT H101N. Surface representation of hinT H101N shown in pink, GMP shown in yellow, GlnA shown in blue.

Figure S5

A.

B.

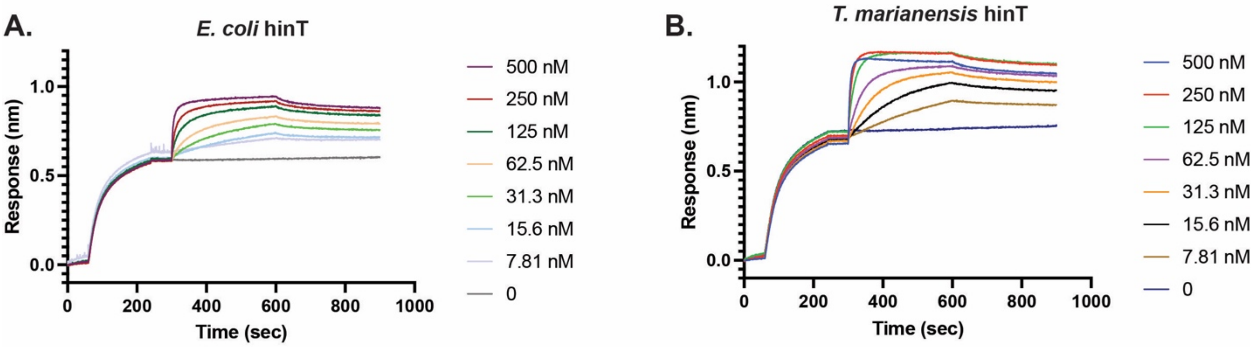

C.

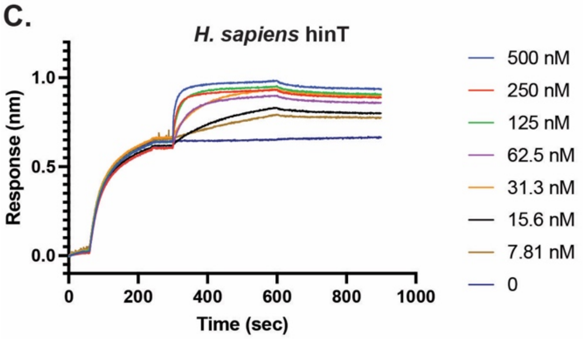

E.

hinT homolog	nM AMP/ μM hinT
E. coli	0.33 ± 0.0939
T. marianensis	0.24 ± 0.0754
H. sapiens	38.68 ± 0.636

D.

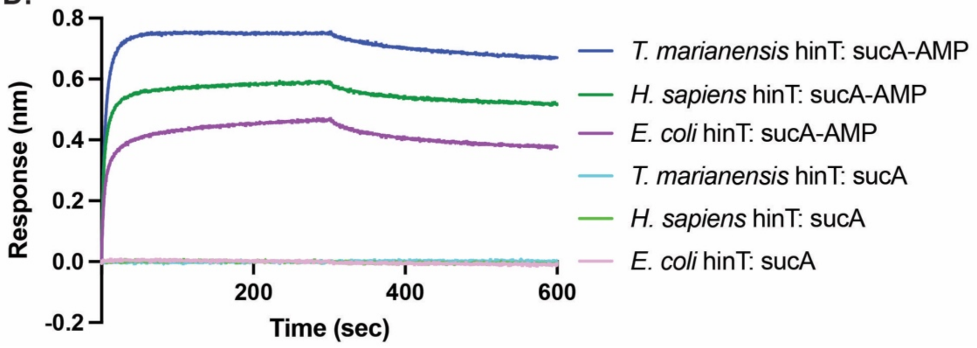

Supplementary figure 5: Binding affinities of hinT homologs to sucA-AMP.

(A-C) Full BLI sensorgrams of the binding response of sucA-AMP to immobilized GST-Ec hinT **(A)**, GST-Tm hinT **(B)**, and GST-Hs hinT **(C)**. Graphs display pin loading with GST-hinT constructs (first response signal increase) followed by sucA binding (second response signal increase).

(D) Representative BLI sensorgrams depicting the binding response of 500 nM AMPylated sucA or unmodified sucA to immobilized GST-*E. coli* hinT, GST-*T. marianensis* hinT, and GST-*H. sapiens* hinT.

(E) AMP nucleotide concentration detected by LC-MS/MS normalized to GST-hinT^{HN} protein concentrations. Data are presented as mean values +/- SD.

Figure S6

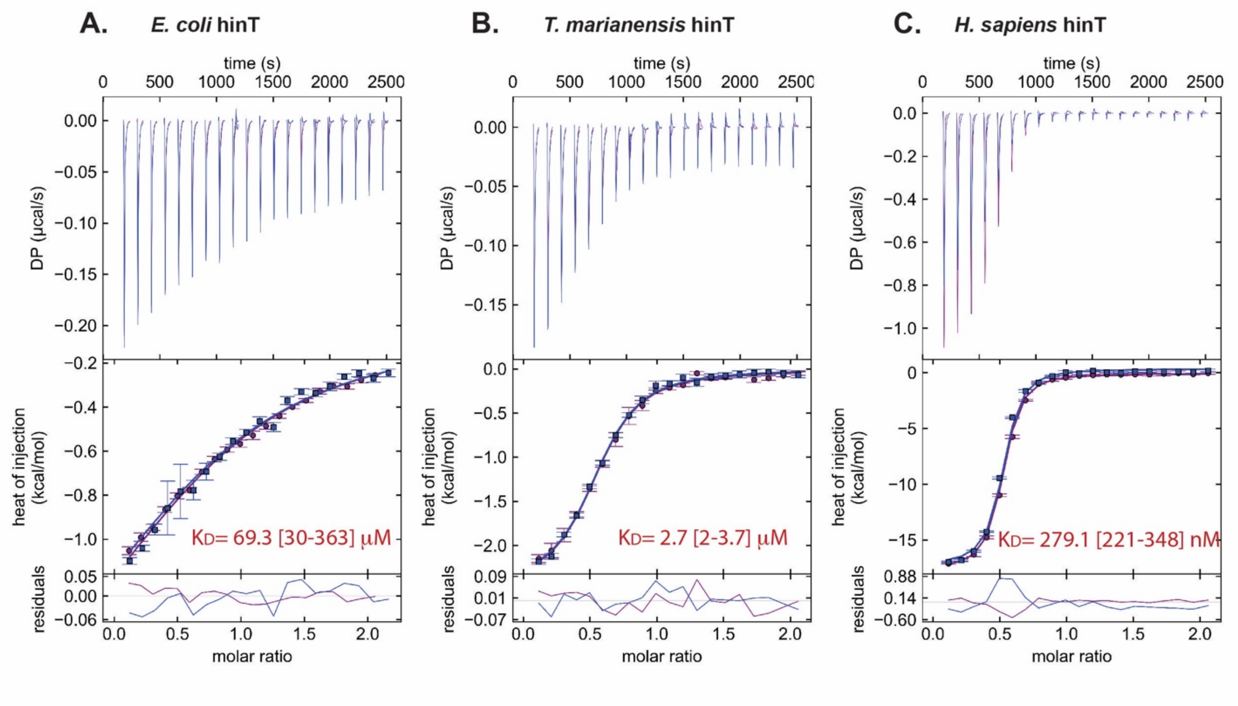

Supplementary figure 6: Binding affinities of hinT homologs to AMP nucleotide.

Isotherms depicting the binding of AMP to GST-hinT homologs. AMP titration to *E. coli* hinT **(A)**, *T. marianensis* hinT **(B)**, and *H. sapiens* hinT **(C)**. Top panels show NITPIC reconstructed thermograms, the middle panels show binding isotherms and individual fits and the bottom panels show the fitting residuals. Dissociation constants (K_D) are obtained from global analysis of duplicate measurements. Reported K_D represents best-fit values with values in brackets indicating the respective 95% confidence interval.

Figure S7

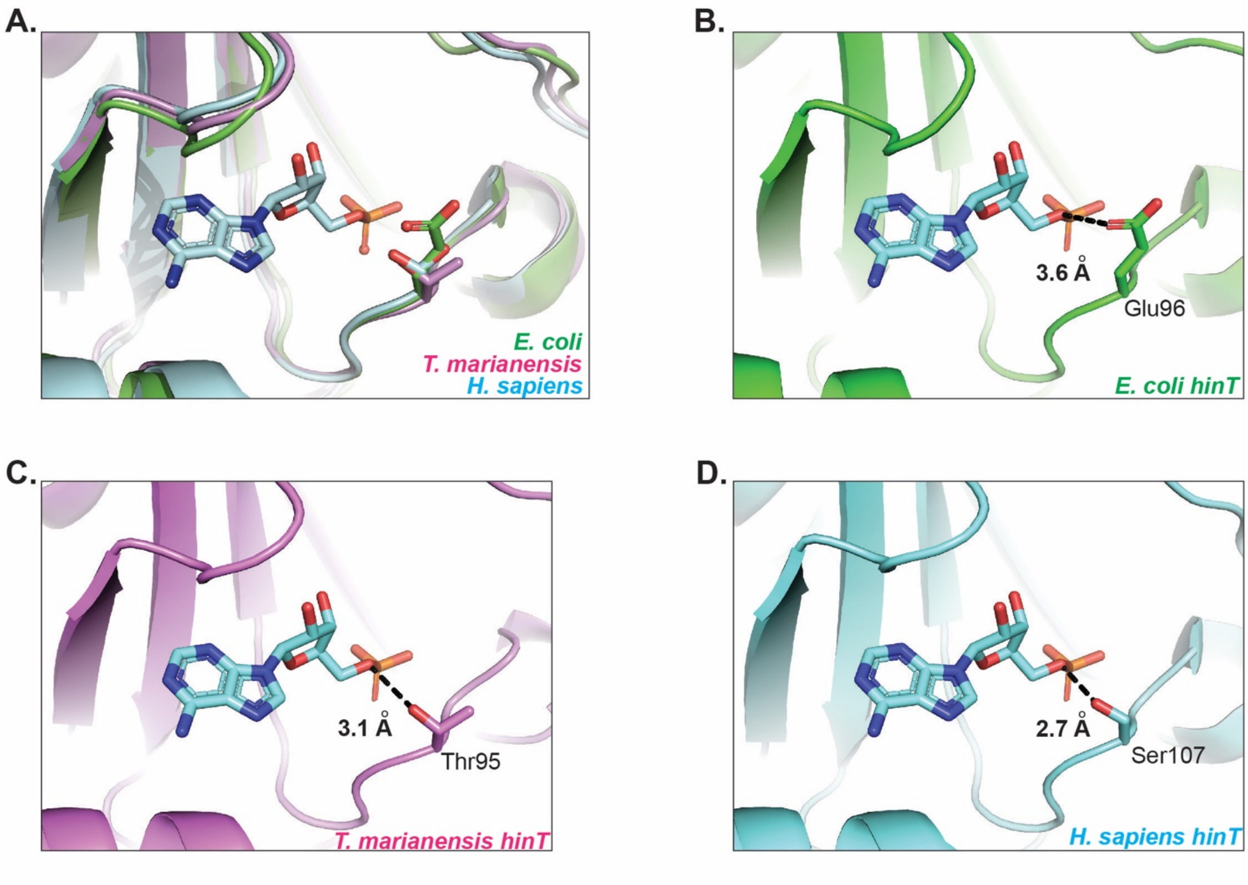

Supplementary figure 7: Structure alignment of nucleotide bound hinT homologs.

- **(A)** Structural alignment of the nucleotide binding pockets of *E. coli* hinT, *T. marianensis* hinT and *H. sapiens* hinT structures bound to purine nucleotide.
- **(B)** Nucleotide binding pocket of *E. coli* hinT (PDB 3N1S). Glutamate 96 is displayed with stick representation and the distance from the phosphate moiety of AMP (from PDB 5KLZ) is shown in Angstrom (Å).
- **(C)** Nucleotide binding pocket of *T. marianensis* hinT (AlphaFold model). Threonine 95 is displayed with stick representation and distance from the phosphate moiety of AMP (from PDB 5KLZ) is shown in Angstrom (Å) and dashed lines.
- **(D)** Nucleotide binding pocket of *H. sapiens* hinT (PDB 5KLZ). Serine 107 is displayed with stick representation and distance from the phosphate moiety of AMP is shown in Angstrom (Å).

Figure S8

A.

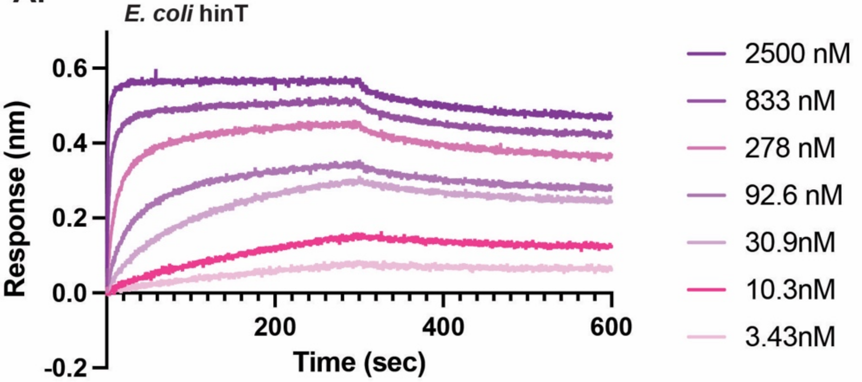

B.

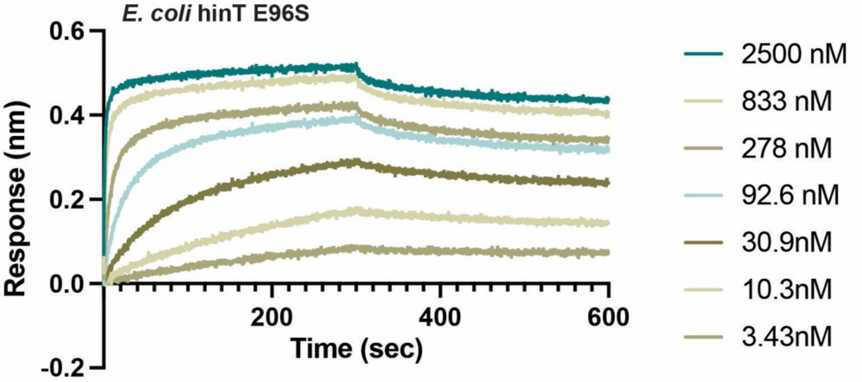

C.

hinT homolog	K_D(nM)	2σ(nM)	R^2
E. coli	30	15 to 58	0.96
E. coli E96S	21	14 to 32	0.98

Supplementary figure 8: Mutation of E96S improves binding affinity of *E. coli* hinT

(A-B) Representative BLI sensorgrams depicting the binding response of serial dilutions 2500 nM to 3.4 nM AMPylated sucA to immobilized GST-*E.*coli hinT (A), GST *E.*coli -hinT^{E96S} (B).

(C) Binding affinities measured from steady state binding response of GST-*E.*coli hinT and GST *E.*coli -hinT^{E96S} to sucA-AMP. The K_D values were determined from

steady state binding responses as a non-linear regression curve and reported with 95% confidence interval.

Figure S9

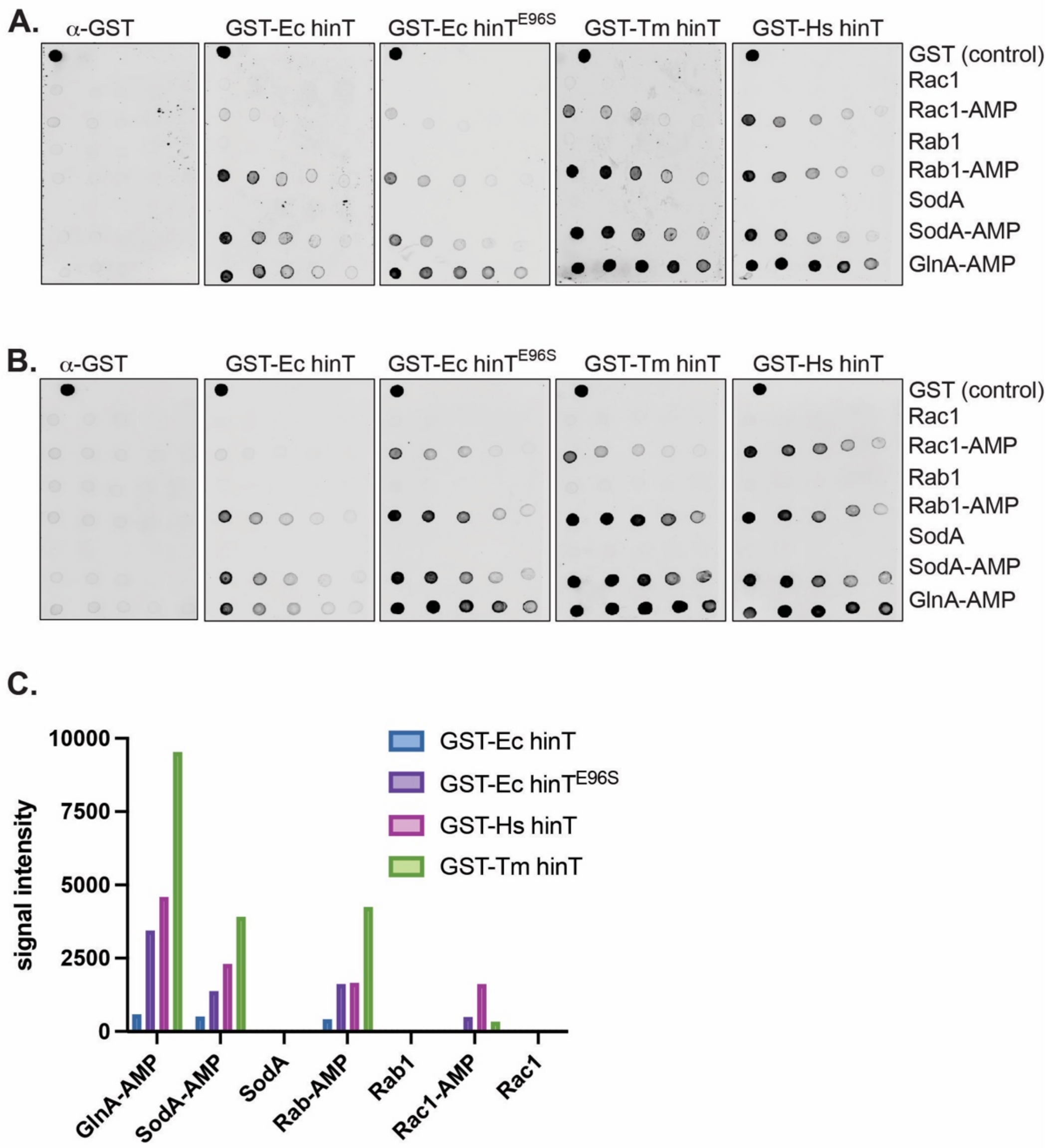

Supplementary figure 9: Dot blot analysis of GST-hinT homologs using AMPylated substrates

(A-B) Independent replicates of dot blot assay represented in Fig 4a. Serial dilutions (400,200,100,50 or 25 ng) of proteins spotted on nitrocellulose membranes and probed with GST, GST-Ec hinT, GST Ec-hinT^{E96S}, GST-Hs hinT or GST-Tm hinT.

(C) Representative bar graph showing signal intensities quantified from 100 ng of proteins spotted in dot blot assays, as shown in Supplementary Fig. 9b.

Figure S10

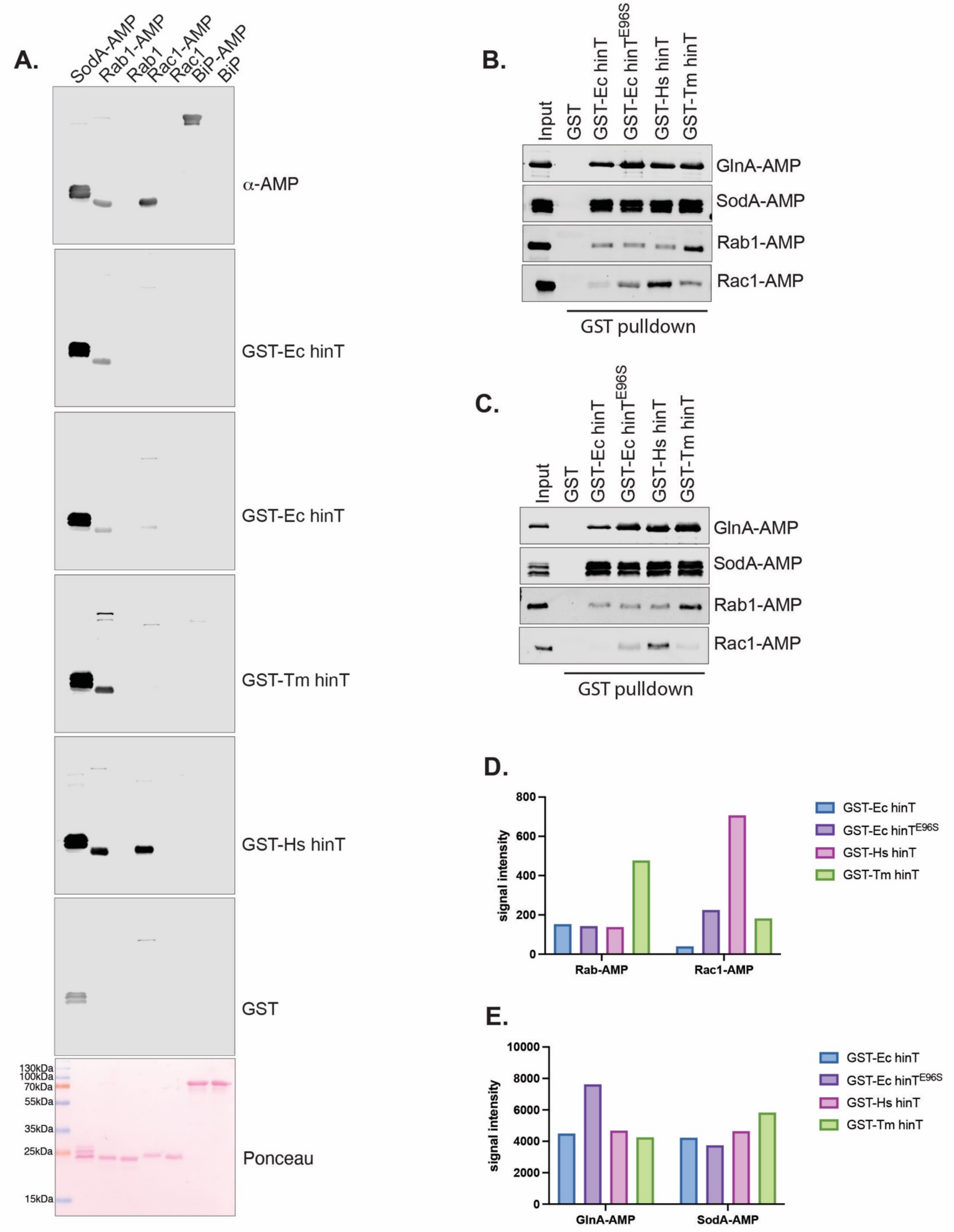

A.
SodA-AMP
Rab1-AMP
Rab1
Rac1-AMP
Rac1
BiP-AMP
BiP
α-AMP
GST-Ec hinT
GST-Ec hinT
GST-Tm hinT
GST-Hs hinT
GST
130kDa
100kDa
70kDa
55kDa
35kDa
25kDa
15kDa
Ponceau
B.
Input
GST
GST-Ec hinT
GST-Ec hinT E96S
GST-Hs hinT
GST-Tm hinT
GlnA-AMP
SodA-AMP
Rab1-AMP
Rac1-AMP
GST pulldown
C.
Input
GST
GST-Ec hinT
GST-Ec hinT E96S
GST-Hs hinT
GST-Tm hinT
GlnA-AMP
SodA-AMP
Rab1-AMP
Rac1-AMP
GST pulldown
D.
signal intensity
800
600
400
200
0
Rab-AMP
Rac1-AMP
GST-Ec hinT
GST-Ec hinT E96S
GST-Hs hinT
GST-Tm hinT
E.
signal intensity
10000
8000
6000
4000
2000
0
GlnA-AMP
SodA-AMP
GST-Ec hinT
GST-Ec hinT E96S
GST-Hs hinT
GST-Tm hinT

Supplementary figure 10: Screening of GST-hinT homologs using AMPylated substrates

- **(A)** Far western analysis of AMPylated substrates using GST, GST-Ec hinT, GST-Ec hinT^{E96S}, GST-Hs hinT or GST-Tm hinT. Protein immunoblot for α-AMP is shown for comparison. Ponceau stained membrane depicts total protein.
- **(B-C)** Independent replicates of pulldown assays depicted in Fig. 4b. α-AMP immunoblotting of enriched proteins from pulldown assay of GST, GST-Ec hinT, GST-Ec hinT^{E96S}, GST-Hs hinT or GST-Tm hinT with AMPylated GlnA, SodA, Rab1 and Rac1. Note that the input for supplementary fig. 10b is 50% while 10c is 10%.
- **(D-E)** Representative bar graph showing signal intensities quantified from the pulldown assay represented in Supplementary Fig. 10b.

Figure S11

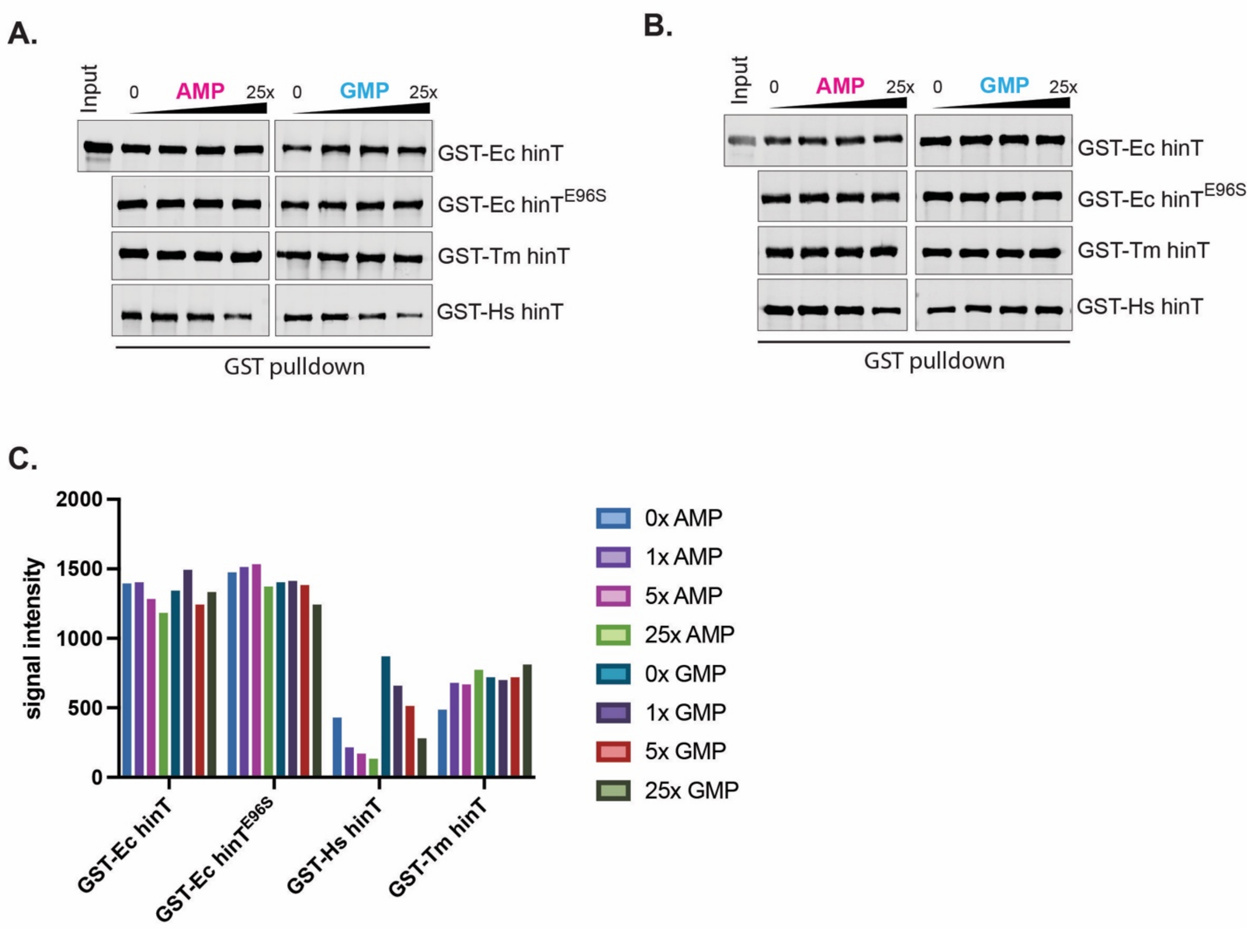

Supplementary figure 11: AMP nucleotide competes with AMPylated proteins for binding to Hs hinT

- **(A-B)** Independent replicates of pulldown assays depicted in Fig. 4c. α-AMP immunoblotting of sucA-AMP bound to GST, GST-Ec hinT, GST-Ec hinT^{E96S}, GST-Hs hinT or GST-Tm hinT in the presence of increasing concentrations of AMP or GMP nucleotides.
- **(C)** Representative bar graph showing signal intensities quantified from the nucleotide competition assay represented in Fig. 4c.

Figure S12

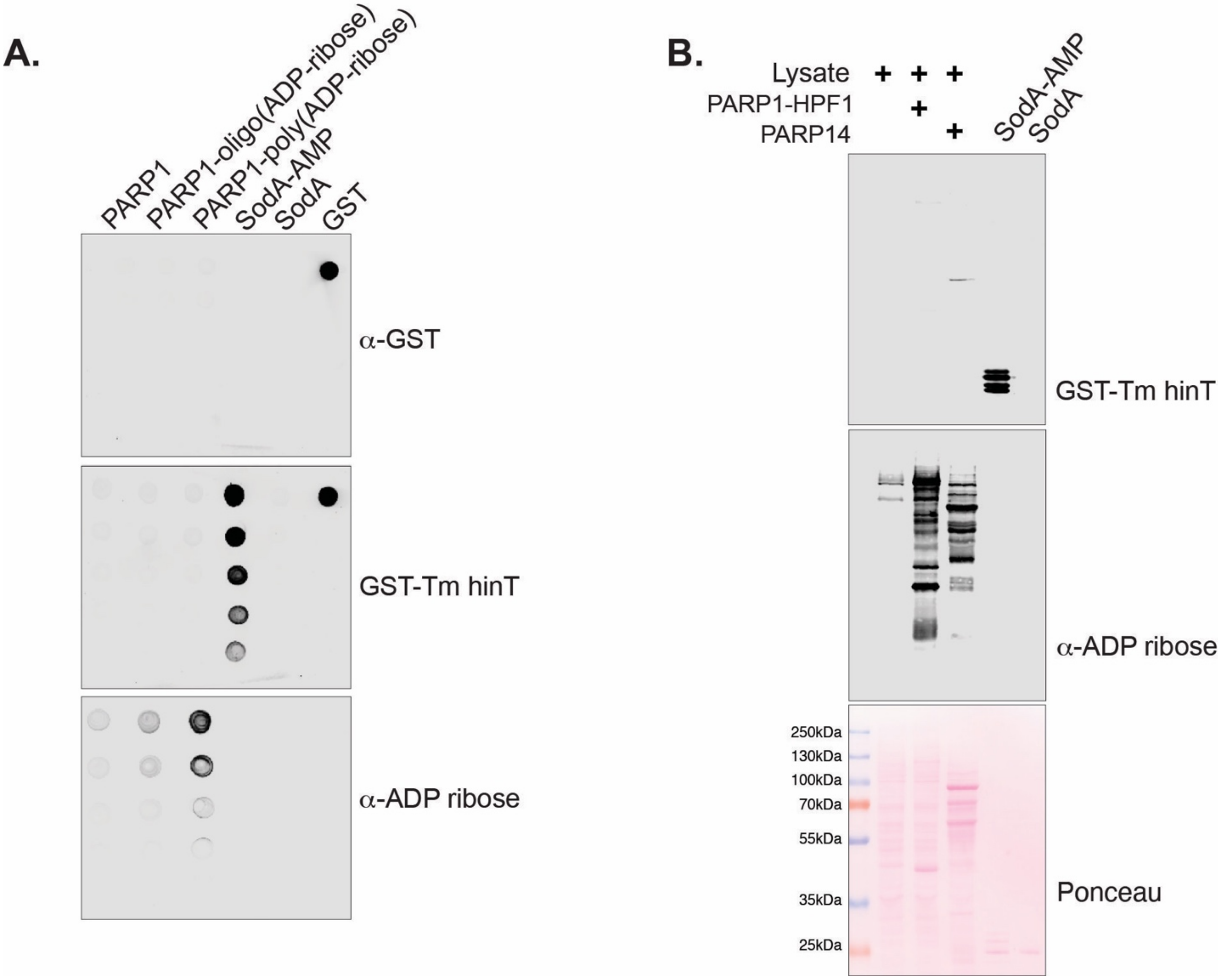

Supplementary figure 12: Tm hinT does not bind ADP-ribosylated proteins

- **(A)** Dot blot assay of AMPylated sodA and auto-ADP ribosylated PARP1 incubated with either GST, GST-Tm hinT or α-ADP ribosylation antibody.
- **(B)** Far-western analysis of AMPylated SodA and ADP ribosylated substrates. Recombinant PARP1/HPF1 or PARP14 were incubated with cell lysates to generate ADP ribosylated proteins in vitro[61]. Membranes were incubated with either GST, GST-Tm hinT or α-ADP ribosylation antibody. GST bound proteins were detected using α-GST antibody.

Figure S13

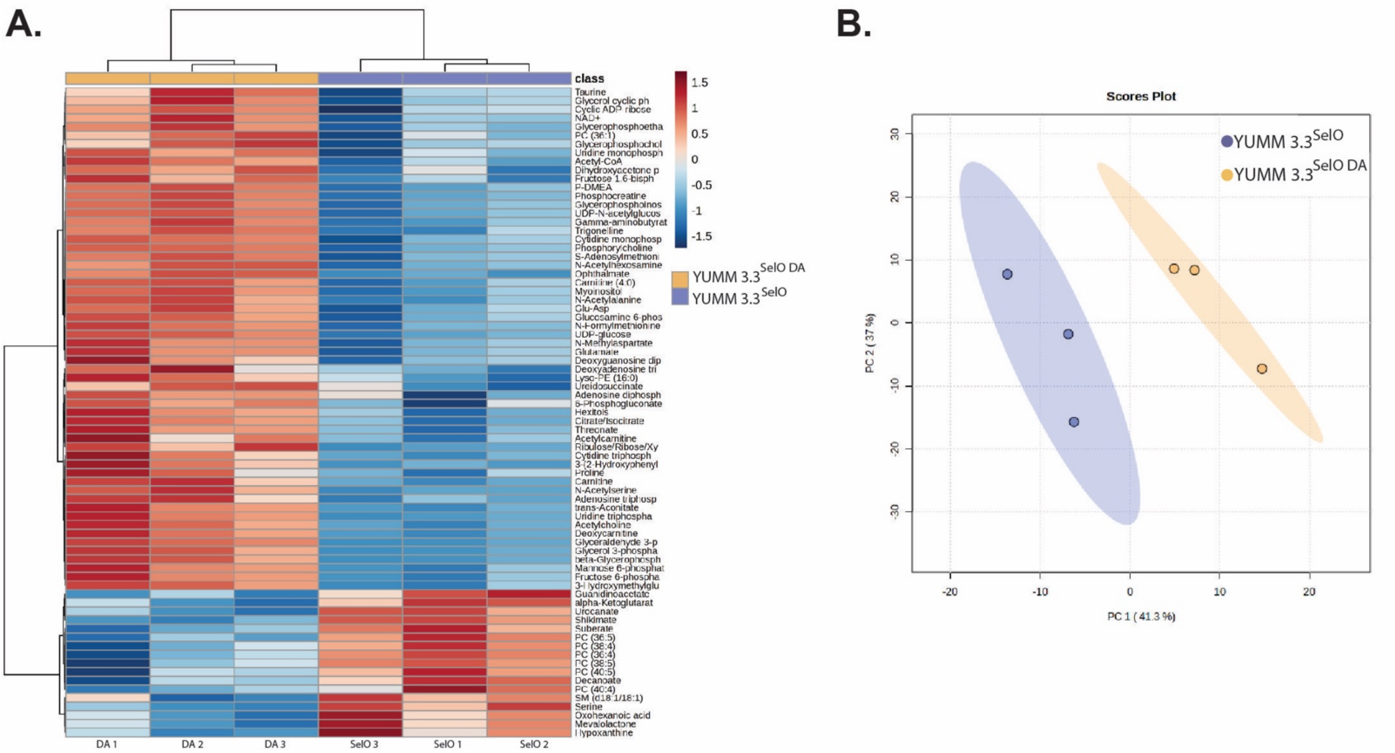

Supplementary figure 13: SelO mediated AMPylation alters metabolite levels in cells

(A) Heat map of top 75 metabolites altered in YUMM 3.3^{SelO} and YUMM 3.3^{D338A}.

(B) Principal component analysis (PCA) plot of metabolites from YUMM 3.3^{SelO} (n=3) or YUMM 3.3^{D338A} (n=3).

Figure S14

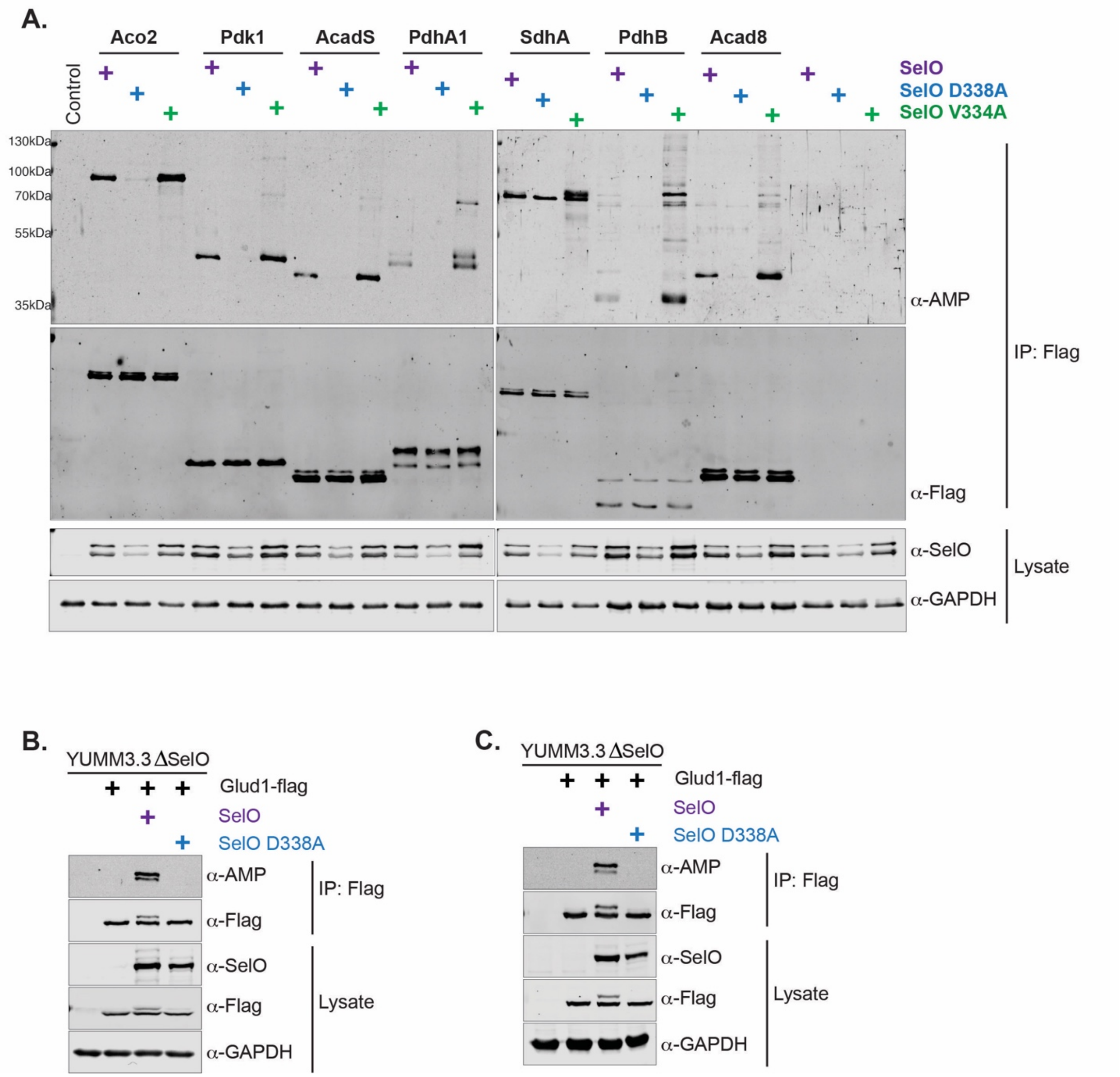

Supplementary figure S14: SelO catalyzes AMPylation of multiple mitochondrial proteins.

(A) Protein immunoblotting of Flag immunoprecipitates or cell lysates from HEK293a cells co-expressing SelO and Flag tagged substrates: mitochondrial aconitase (aco2), pyruvate dehydrogenase kinase (pdk1), short-chain specific acyl-CoA dehydrogenase (acadS), pyruvate dehydrogenase E1 component subunit α (pdhA1), succinate dehydrogenase flavoprotein subunit (sdhA), pyruvate

dehydrogenase E1 component subunit β (pdhB), isobutyryl-CoA dehydrogenase (acad8). Control (lane 1) denotes non-transfected HEK293a cells.

(B) Protein immunoblotting of flag immunoprecipitates or cell lysates from YUMM3.3ΔSelO expressing Glud1-Flag or Glud1-Flag and SelO or $SelO^{D338A}$. Results are representative of at least 3 independent experiments (related to Fig. 6d). First lane denotes YUMM3.3ΔSelO non-transduced parental cells.

Figure S15

A.

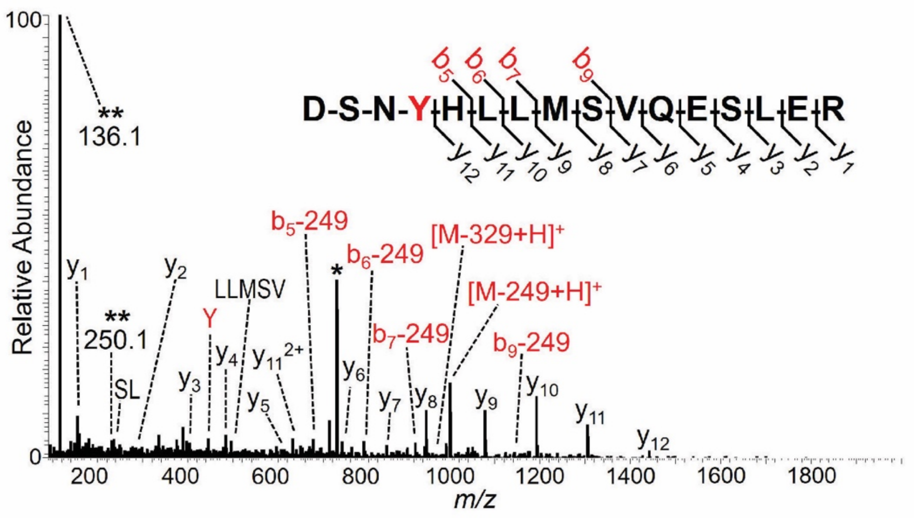

B.

		Spectral Count	
**Modified Residue**	**Glud1 Peptide**	**AMPylated**	**unmodified**
Y464	DSN**Y**HLLMSVQESLER	39	48
Y528	**Y**NLGLDLR	1	52
Y550	V**Y**NEAGVTFTLESR	1	127

Supplementary figure 15: Mass spectrometry analysis of Glud1

(A) Representative MS/MS spectra of AMPylated Glud1 peptide ions DSNYHLLMSVQESLER. The precursor ion is labeled with an asterisk (*) and HCD fragmentation generated b- and y- fragment ions shown. Ions highlighted in red contain a mass shift associated with neutral loss of the AMP group. Neutral loss products are also present at 136.1 and 250.1 Da (**) in AMPylated spectra. Any fragment ions labeled with (*) correspond to loss of H2O (-18 Da) or NH3 (-17 Da).

(B) Comparison of AMPylated versus unmodified peptide spectral counts identified by MS/MS analysis of Glud1 proteins immunoprecipitated from YUMM 3.3^{SelO}. AMPylation sites are highlighted in red. Spectral counts reflect the number of MS2 peptide spectral matches as determined by Mascot software searches.

Figure S16

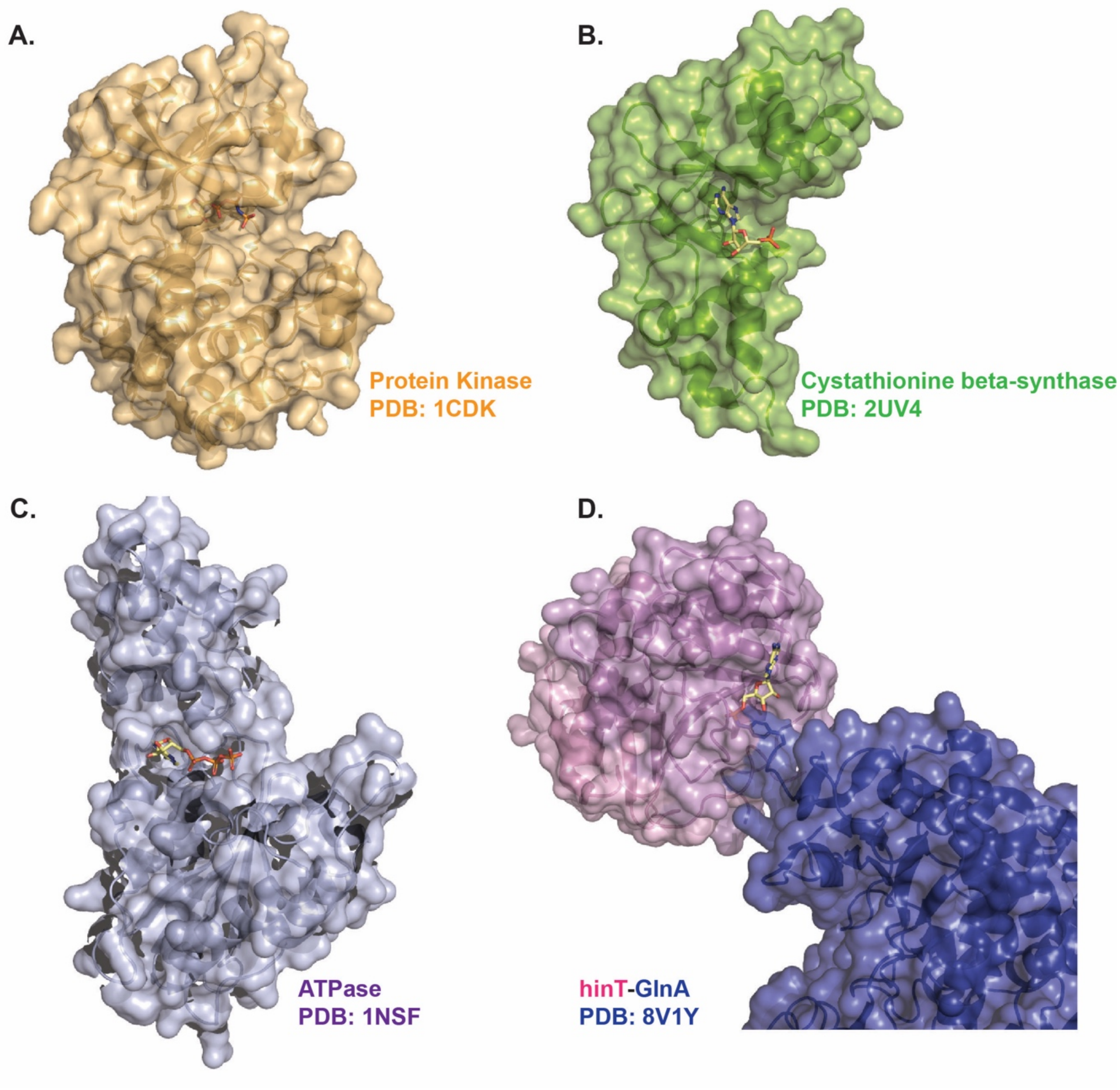

Supplementary figure 16: Comparison of binding pockets in nucleotide-binding proteins

Surface representation of nucleotide binding domains: protein kinase shown in orange, cystathionine-beta synthase shown in green, ATPase in light purple, hinT in pink, GlnA in blue. Stick representation of nucleotide is shown in yellow.

(A) Catalytic subunit of protein kinase A (PKA) in complex with AMP-PNP (PDB: 1CDK). AMP-PNP is shown in stick representation

(B) Cystathionine beta-synthase (CBS) domain from AMPK gamma1 subunit in complex with AMP (PDB: 2UV4). AMP is shown in stick representation

(C) D2 ATPase domain of N-ethylmaleimide-sensitive factor (NSF) in complex with ATP (PDB: 1NSF). ATP is shown in stick representation.

(D) hinT in complex with GlnA-AMP (PDB: 8V1Y).

Figure S17

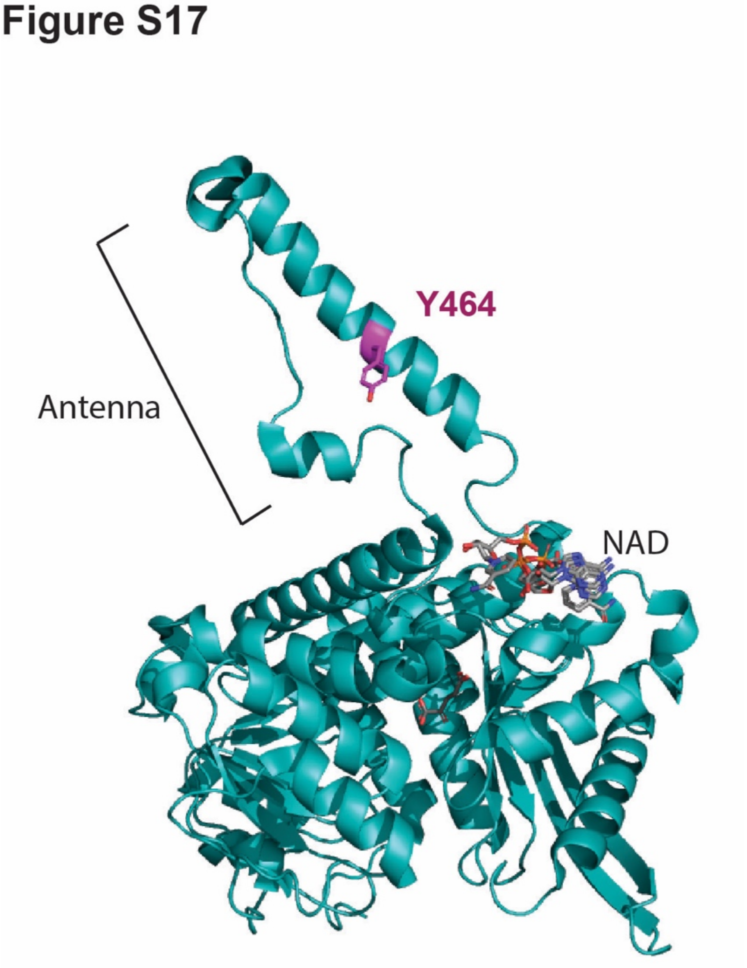

Supplementary figure 17: AMPylated Glud1 can be enriched using hinT

Ribbon representation of bovine Glud1 (PDB: 1HWY). Putative site of AMPylation, Y464, as revealed by MS analysis shown in magenta. NAD is shown in stick representation.

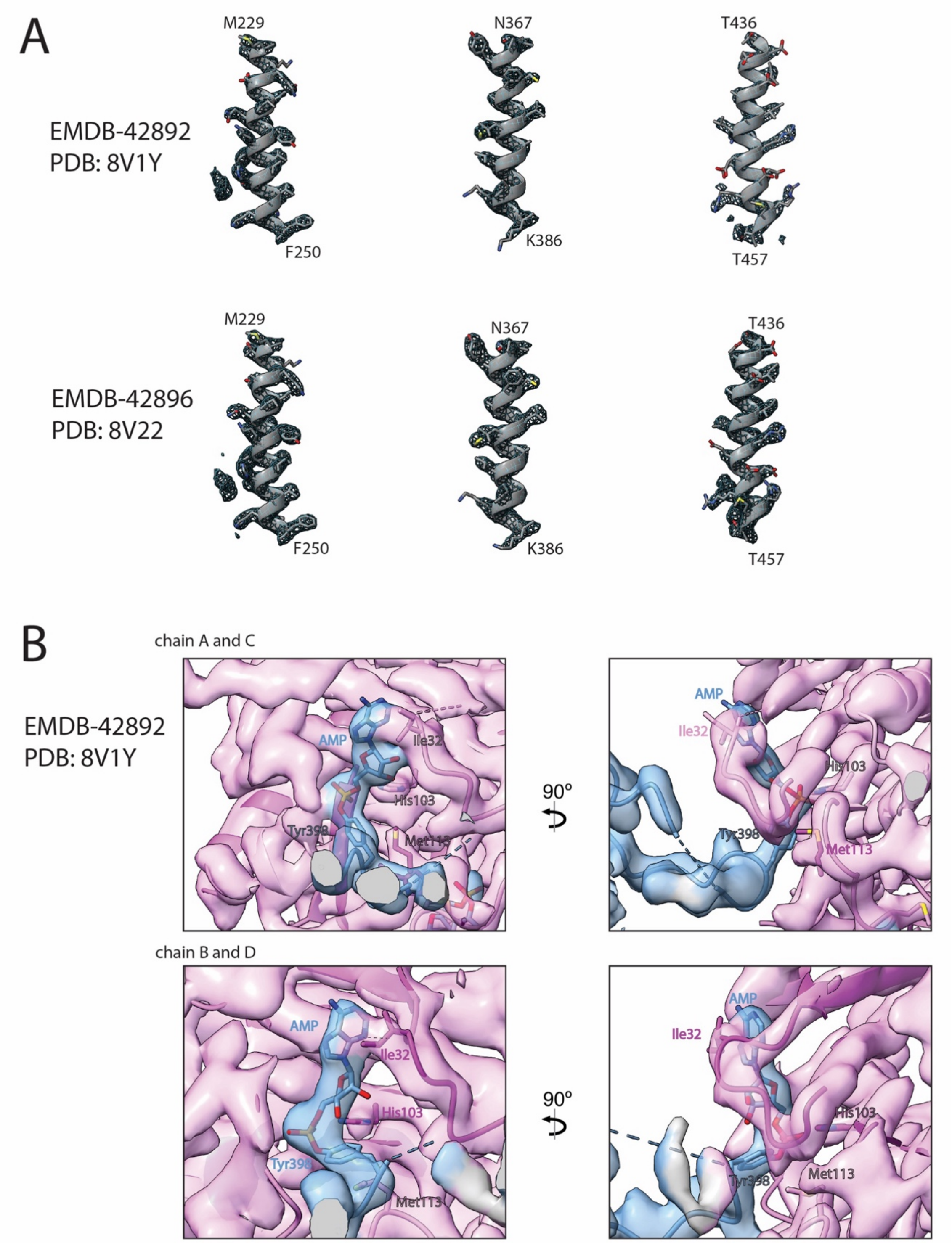

Supplementary figure 18. Model-map fit.

(A) Representative α helices showing model-map fit. Upper panel is for the composite map (EMDB-42892) fitted with PDB 8V1Y, map shown as a mesh at threshold level

0.35; lower panel is for the dodecamer map (EMDB-42896) fitted with PDB 8V22, map shown as a mesh at threshold level 0.25.

(B) Model-map fit of AMP. Composite map (EMDB-42892) fitted with PDB 8V1Y. Map was shown as a transparent surface at threshold level 0.35. Map and model were colored similar to Figure 2C. Upper panels show two views of AMP between chain A and C in 8V1Y; lower panels show two views of AMP between chain B and D in 8V1Y.

Supplemental Tables

Uniprot Accession	Protein Description	Protein Ion Score	PSMs	Unique Peptides
**P0A9C5**	**Glutamine synthetase OS=Escherichia coli (strain K12)**	**177.576**	**1204**	**37**
C5A1D5	Chaperonin GroEL OS=Escherichia coli (strain K12 / MC4100 / BW2952)	137.442	181	31
P0A9P0	Dihydrolipoyl dehydrogenase OS=Escherichia coli (strain K12)	92.145	75	26
P25553	Lactaldehyde dehydrogenase OS=Escherichia coli (strain K12)	54.329	49	17
P0AD61	Pyruvate kinase I OS=Escherichia coli (strain K12)	49.628	32	14
C4ZZ12	ATP synthase subunit alpha OS=Escherichia coli (strain K12 / MC4100 / BW2952)	36.861	31	17
P0ABB4	ATP synthase subunit beta OS=Escherichia coli (strain K12)	34.419	39	7
B1XFM4	Trigger factor OS=Escherichia coli (strain K12 / DH10B)	33.863	18	12
P33224	Putative acyl-CoA dehydrogenase AidB OS=Escherichia coli (strain K12)	30.967	23	10
B1XC24	Glucose-6-phosphate isomerase OS=Escherichia coli (strain K12 / DH10B)	27.591	15	11

Supplementary Table 1. Top 10 hits from Protein ID search using Proteome Discoverer 2.2 software.
MS/MS spectra were searched against sequences in the Uniprot Escherichia coli (strain K12) protein database (Taxon ID 83333). PSMs = peptide-spectrum matches (MS/MS spectral count)

	GlnA Dodecamer (EMDB: 42896) (PDB: 8V22)	GlnA dimer with hinT dimer (EMDB: 42895)	hinT focused map (EMDB: 42894)	Composite map (EMDB: 42892) (PDB: 8V1Y)
**Data collection and processing**				
Magnification	105,000			
Voltage (kV)	300			
Electron exposure (e^{-}/Å^{-2})	60			
Defocus range (μm)	-0.9 to -2.2			
Pixel size (Å)	0.83			
Symmetry imposed	C6	C1	C1	
Initial particle images (no.)	989,142	1,139,875	225,422	
Final particle images (no.)	495,703	225,422	225,422	
Map resolution (Å)	2.2	2.7	3.5	
FSC threshold	0.143	0.143	0.143	
Map resolution range (Å)	2.2-	2.69-	3.46-	
Map pixel size (Å)	0.83	0.83	0.83	
**Refinement**				
Initial model used (PDB code)	7W85			7W85, 3N1T
Model composition				
Nonhydrogen atoms	42,195			8,918
Protein residues	5,439			1,137
Ligands	12			2
R.m.s. deviations				
Bond lengths (Å)	0.003			0.002
Bond angles (°)	0.490			0.461
Validation				
MolProbity score	1.02			1.28
Clashscore	2.32			5.27
Poor rotamers (%)	0			0
Ramachandran plot				
Favored (%)	97.94			98.2
Allowed (%)	2.06			1.8
Disallowed (%)	0			0

Supplementary Table 2: Cryo-EM data collection, refinement, and validation statistics.

Name	Sequence
Ec hinT_BamHI Fwd	AAAA GGATCC ATGGCAGAAGAAACTATATTCAG
Ec hinT_XhoI Rev	AAAA CTC GAG TTACAGACCTTTATGCGCC
Hs Hint1_BamHI Fwd	AAAA GGATCC ATGGCAGATGAGATTGCCA
Hs Hint1_XhoI Rev	AAAA CTC GAG TTAACCAGGAGGCCAATGC
Tm hinT Bam Fwd	AAAA GGATCC ATGGCGGACTGCCTG
Tm hinT Xho Rev	AAAA CTC GAG TCAACCGGGAGGCCAC
Ec SelO_V242A Fwd	gttgtcggtattcatcgccccatgagcaaagcc
Ec SelO_V242A Rev	ggctttgctcatggggcgatgaataccgacaac
EcHint E96S Fwd	ggatgtggtaaaccgattgtccgccatggcggttg
EcHint E96S Rev	caaccgccatggcggacaatcggtttaccacatcc
hinT H101N Fwd	cacccaacaagtgcatattgatgtggtaaacctcttg
hinT H101N Rev	caagaggtttaccacatcaatatgcacttgttgggtg
HsHint1 H112N Fwd	ctccaagaacatggagattaacgtgatagacagactgt
HsHint1 H112N Rev	acagtctgtctatcacgttaatctccatgttcttggag

Supplementary Table 3: Oligonucleotides used in this study
