# Supplementary material for: A repurposed AMP binding domain reveals mitochondrial protein AMPylation as a regulator of cellular metabolism
Source: Nat Commun. 2025 Aug 23;16:7863. doi: 10.1038/s41467-025-63014-z (PMC12375108; doi:10.1038/s41467-025-63014-z)
Supplement: Supplementary file 2 — Description of Supplementary files [file 41467_2025_63014_MOESM2_ESM.docx]

**Description of Supplementary files**

**Supplementary Figures**: Figures S1-S18

**Supplementary Table 1**: Protein identification of AMPylated proteins by mass spectrometry analysis.

**Supplementary Table 2**: Data collection and refinement statistics of GlnA-hinT H101N complex.

**Supplementary Table 3**: Oligonucleotides used in this study

**Supplementary Data 1**: Excel file of AMPylated proteins enriched using GST-hinT H101N from *E. coli* over expressing WT SelO or the hyperactive V242A SelO.

**Supplementary Data 2**: Excel file of AMPylated proteins enriched using GST-hinT H101N from YUMM3.3 over expressing SelO or inactive mutant D338A SelO. P-values were calculated using an ANOVA test.

**Supplementary Data 3:** Excel file of raw metabolomics data related to Figure 6a**.** P-values were calculated using t-test.
